# Neuroblastoma: Anti‐Invasive Effects of Tetracaine In Vitro*

**DOI:** 10.1111/bcpt.70190

**Published:** 2026-02-01

**Authors:** Ece Selçuk, David S. Silcock, Amanda Foust, Vicky Bousgouni, Mar Arias‐Garcia, Chris Bakal, Mustafa B. A. Djamgoz, Pinar Uysal‐Onganer

**Affiliations:** ^1^ Cancer Mechanisms and Biomarkers Research Group, School of Life Sciences University of Westminster London UK; ^2^ Department of Molecular Biology and Genetics, Faculty of Engineering and Natural Sciences Istanbul Medeniyet University Istanbul Türkiye; ^3^ Departments of Bioengineering Imperial College London, South Kensington Campus London UK; ^4^ Dynamical Cell Systems Team, Division of Cancer Biology The Institute of Cancer Research‐Chester Beatty Laboratories London UK; ^5^ Departments of Life Sciences Imperial College London, South Kensington Campus London UK

**Keywords:** invasion, metastasis, neuroblastoma, tetracaine, voltage‐gated sodium channel

## 1 Introduction and Background

Neuroblastoma (NB), a neural crest‐derived tumour, is one of the most common extracranial solid malignancies in children. Most patients are under 5 years old at diagnosis (median, 18 months) [1]. The main cause of death is metastasis with high‐risk patients having a survival rate below 50% over 5 years. The main treatment for high‐risk cases is multimodal involving surgery, chemotherapy, radiotherapy, immunotherapy and stem cell transplantation. However, despite aggressive therapy, more than 50% of children with high‐risk NB relapse. Consequently, there is significant unmet need in diagnosing metastatic NB early and treating it effectively and, ideally, non‐toxically [1].

Here, we aimed to elucidate the potential anti‐invasive role of tetracaine. Tetracaine is a well‐established blocker of voltage‐gated sodium channel (VGSC) activity shown previously to promote metastatic cell behaviours in several cancers [2, 3]. It is in routine clinical use as a long‐acting local anaesthetic [4]. Several human NB cell lines have been shown to express VGSCs [5, 6]. Indeed, a novel developmentally regulated (‘neonatal’) splice variant of Nav1.5 was first described in a NB cell line [5]. However, little is known about the possible pathophysiological role of VGSC expression in NB.

## 2 Materials and Methods

The study adhered to the *Basic and Clinical Pharmacology and Toxicology* policy for experimental and clinical studies [7]. All the procedures used, except the following, have been described already [2].

### 2.1 Bioinformatics

Transcriptomic expression data were extracted from available databases [8, 9]. For non‐normalised data, mRNA transcript abundances were normalised relative to all the detected transcripts in the sample. This was done by calculating the relative abundance of each gene transcript relative to the total amount of mRNA in each sample, accounting for the fact that the number of sequencings reads per sample may vary. For the housekeeping genes *RPL9* and *ELAVL1*, there was less than a twofold difference in expression in the different cell lines. For the glutamate receptor associated *GRINA*, there was a 2.3‐fold variability in expression. The expression levels were lower and more varied for all the VGSC genes, so a threshold of 0.5 was applied to determine the relative levels of expression across the NB cell lines.

### 2.2 Cell Culture and Pharmacology

In a comparative approach, two of the cell lines with markedly different invasiveness were adopted: SH‐EP cells, characterised as non‐aggressive with low invasive potential, and SK‐N‐SH cells, representing a more aggressive phenotype. The cells were grown in high‐glucose Dulbecco's modified Eagle's medium (DMEM) supplemented with 10% foetal bovine serum (FBS) and 1% penicillin/streptomycin (Gibco, Thermo Scientific, USA). Cells were maintained at 37°C in a humidified 5% CO_2_ incubator. Tetracaine (Thermo Scientific, USA) was prepared in ethanol as a 50 mM stock solution and stored at −20°C until use. Culture media containing equimolar solvent was used as controls.

### 2.3 Data Analysis

Data were analysed as means ± standard errors of the mean. Statistical analyses were performed with GraphPad Prism 8 software (GraphPad Software, USA). Each experiment was performed independently three times. Significance was evaluated using one‐way ANOVA for multiple comparison and two‐tailed unpaired Student's *t*‐test for two‐group comparison. Significance was denoted as ‘*’ (*p* < 0.05) or ‘**’ (*p* < 0.01).

## 3 Results

Six different human NB cell lines were adopted: SH‐EP, IMR‐5, SH‐SY5Y, KELLY, SK‐N‐BE and SK‐N‐SH.

### 3.1 Initial Observations

Transcriptomic profiling revealed stable expression of housekeeping genes (*RPL9* and *ELAVL1*), validating their use in normalisation, whereas *GRINA* (encoding for an ionotropic glutamate receptor) displayed considerable variability. Among VGSCs, *SCN8A* (Nav1.6) was expressed ubiquitously (Table 1). By contrast, *SCN5A* (Nav1.5) expression exhibited a restricted profile. It was undetectable in SH‐EP and SH‐SY5Y but highly expressed in SK‐N‐SH cells. Thus, Nav1.5 expression could underlie a part of the heterogeneity of NB [8, 9]. Of the two contrasting cell types adopted for the experiments, the SK‐N‐SH cell line demonstrated an overall neuroblast phenotype (N‐type). These cells had neurite‐like processes, reflecting their partially differentiated neuronal characteristics. In contrast, SH‐EP cells, derived from the substrate‐adherent (S‐type) subpopulation of SK‐N‐SH cells, lacked most neuronal characteristics (including neurites), displayed a flattened, epithelial‐like morphology with a fibroblast‐like appearance. They adhered tightly to the culture surface and grew as a monolayer. These differences made SH‐EP a useful model of non‐aggressive/less invasive NB, whereas SK‐N‐SH cells served as a model with relatively strong metastatic potential.

**TABLE 1:** Essential characteristics of the NB cell lines used in the study.

Cell line	Background molecular characteristics	Basic pathophysiological characteristics	VGSC α‐subunit mRNA expression
SH‐EP	Non‐MYCN‐amplified Epithelial‐like clone of SK‐N‐SH cell line p53 wt S‐type	Derived from a 4‐year‐old female patient's bone marrow metastasis Intrinsically non‐tumourigenic; lacking processes Doubling time = 30–40 h Substrate‐adherent/non‐invasive	Na_v_1.6 > Na_v_1.2 > Na_v_1.1
IMR5	MYCN‐amplified p53 wt N‐type	Derived from a young child's untreated tumour Adherent growth Doubling time = 20–30 h Invasive	Na_v_1.7 > Na_v_1.6 > Na_v_1.5 > Na_v_1.3
SH‐SY5Y	Non‐MYCN‐amplified p53 wt N‐type	Neuroblast‐like thrice‐clone of SK‐N‐SH (derived from bone marrow biopsy of a 4‐year‐old girl) Large, flat, epithelial‐like phenotype with many short processes Exhibit immature neuronal characteristics Doubling time: 48–60 h Non‐invasive	Na_v_1.7 > > Na_v_1.3 > Na_v_1.2 > Na_v_1.6 > Na_x_
KELLY	MYCN‐amplified p53 mutant	Derived from the tumour of a 1‐year‐old female patient Adherent, capable of growth in monolayers Doubling time = 30–40 h Invasive	Na_v_1.6 > Na_v_1.1 > Na_v_1.3 > Na_v_1.5 > Na_v_1.7 > Na_v_1.2
SK‐N‐BE	MYCN‐amplified p53 mutant	Derived from bone marrow biopsy of a 22‐month‐old male patient Display variable morphology, form loosely adherent aggregates Stem cell–like properties Doubling time = 20–30 h Invasive	Na_v_1.7 > Na_v_1.3 > Na_v_1.2 > Na_v_1.6 > Na_v_1.5
SK‐N‐SH	Non‐MYCN‐amplified p53 wt I‐type	Derived from a metastatic bone marrow aspirate Comprises two morphologically distinctive cell types, a small spiny cell and a large epithelioid cell Doubling time: 40–50 h Highly invasive	Na_v_1.3 > Na_v_1.2 > Na_v_1.5 > Na_v_1.6 > Na_v_1.7

*Note:* S‐type: Substrate‐adherent type of NB cell lines; generally less aggressive. N‐type: Neuroblastic type of NB cell lines; generally more aggressive. I‐type: Intermediate type of NB cell lines, described as a progenitor of N‐ and S‐types.

### 3.2 Tetracaine Had no Effect on Cell Viability or Proliferation

**FIGURE 1:**
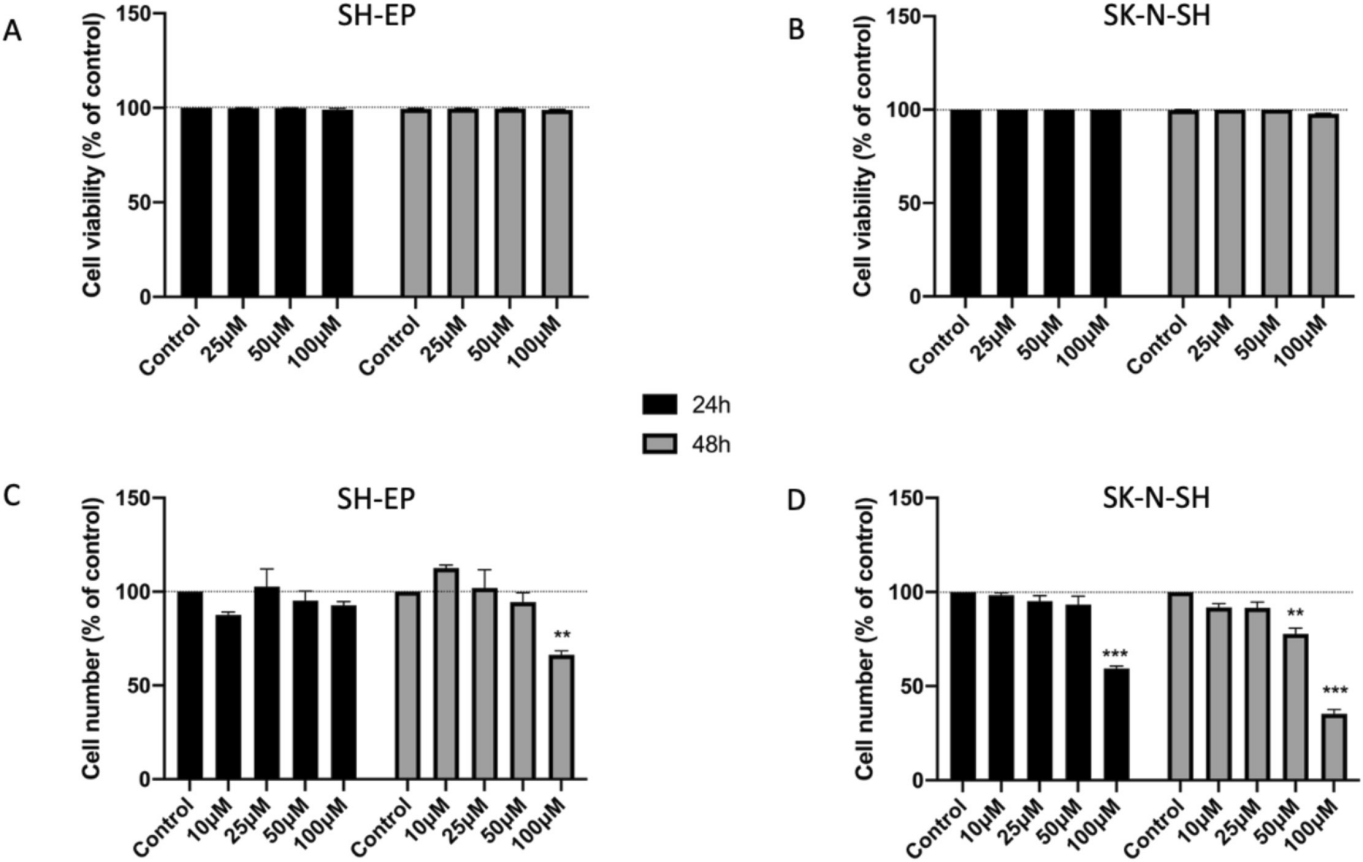
Effects of tetracaine on cell viability and proliferation. Cell viability (%) after treatment with 25, 50 and 100 μM tetracaine for 24 and 48 h for (A) SH‐EP and (B) SK‐N‐SH cells. (C,D) Relative cell numbers (%) from MTT assay involving following treatment with tetracaine (10, 25, 50 and 100 μM) for 24 and 48 h for (C) SH‐EP and (D) SK‐N‐SH cells. Bars denote means and SEMs; **p < 0.01; ***p < 0.001 (*n* = 3). The inset denoting data for 24 and 48 h are applicable to all panels.

Treatment of both cell lines for 24–48 h with tetracaine up to 100 μM had no effect on their viability (Figure 1A,B). Moreover, tetracaine did not exhibit any noticeable effect on the proliferation of SH‐EP cells at concentrations up to 100 μM, or of SK‐N‐SH cells up to 50 μM (Figure 1C,D). In the following experiments, accordingly, the working concentrations of tetracaine were fixed as 50 μM for SH‐EP and 25 μM for SK‐N‐SH cells, so as to ensure viability of the cells and no effect on proliferative activity while studying motility and invasiveness. Such limited effects of tetracaine are similar to previous observations on human breast, prostate and colon cancer cells using tetrodotoxin (TTX) as a highly specific blocker of VGSCs [2].

### 3.3 Tetracaine Inhibited Metastatic Cell Behaviours

**FIGURE 2:**
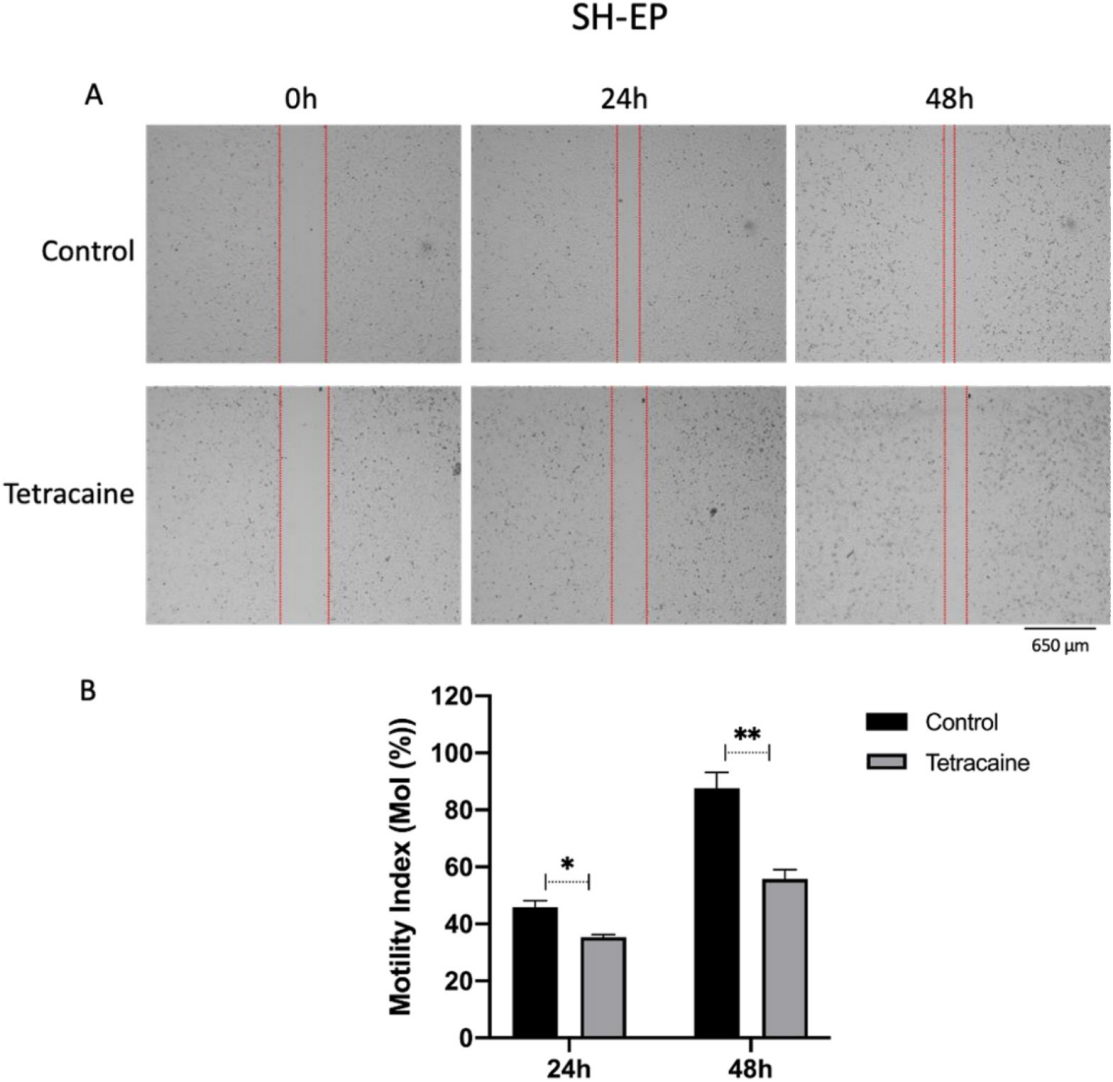
Effect of tetracaine (50 μM) on lateral motility of SH‐EP cells measured in scratch assays over 24–48 h. Motility was quantified as the ‘Motility Index’ (defined in Methods section). (A) Representative images of the wounds taken at the start (0 h) and 24 and 48 h post‐scratch. Scale bar applies to all panels. (B) Quantification of motility data from (A), performed using ImageJ. Bars denote means and SEMs; *p < 0.05; **p < 0.01 (*n* = 3).

Two types of cell behaviour involved in the metastatic cascade were studied: lateral motility and Matrigel invasion. Lateral motility resembles early stages of metastatic cascade when the tumour cells are moving out of their primary sites, whereas Matrigel invasion represents more advanced behaviour involving both motility and proteolytic degradation of the extracellular matrix. Preliminary experiments revealed that SH‐EP cells were not invasive. However, the cells were motile. Thus, in scratch assays, their motility index (MoI) increased significantly from 0.45 (24 h) to 0.87 (48 h). Treatment with tetracaine (50 μM) inhibited lateral motility, MoI falling by 10% and 35% after 24 and 48 h, respectively (Figure 2). These effects were statistically significant (*p* < 0.05 and < 0.01).

**FIGURE 3:**
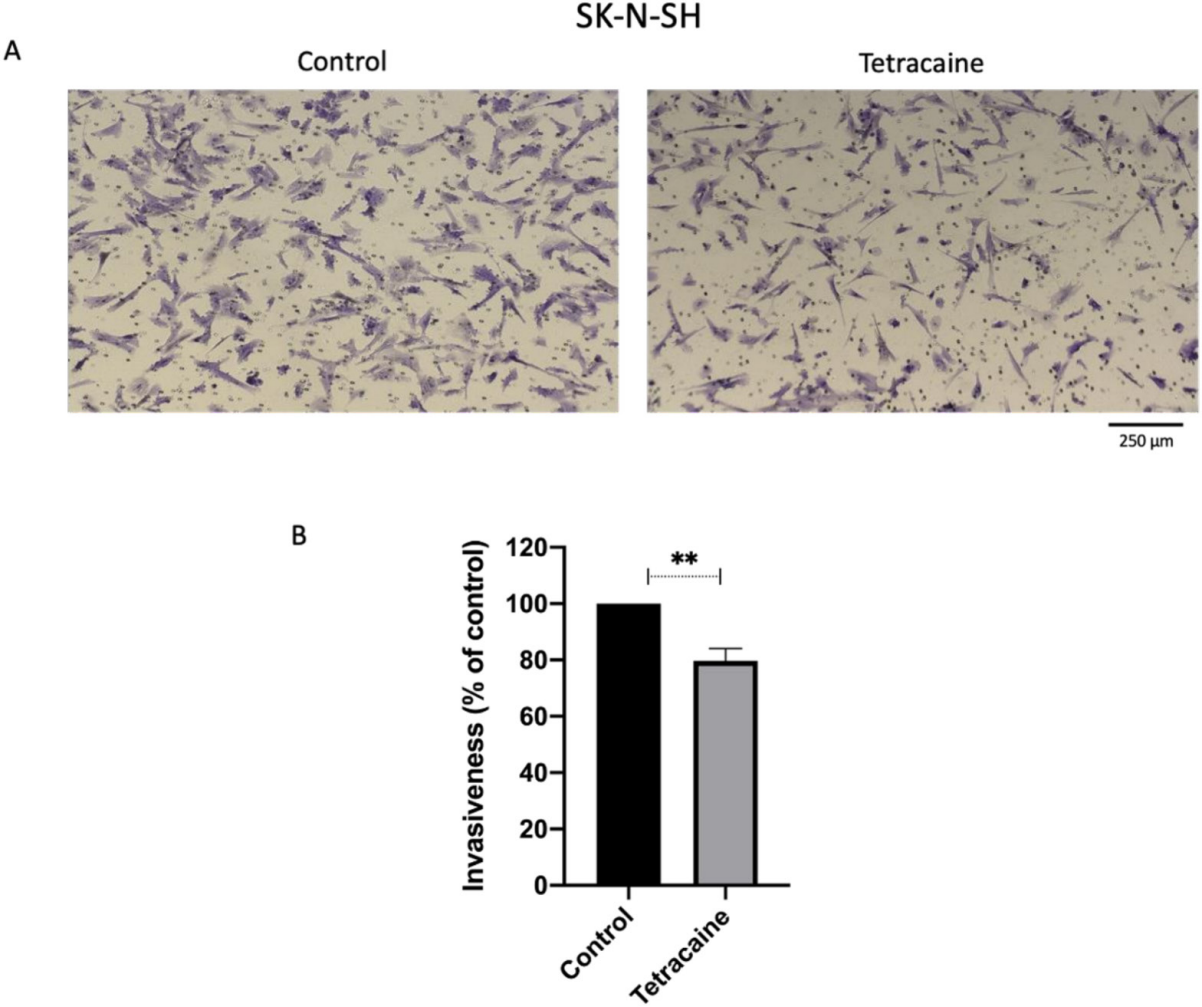
Anti‐invasive effect of tetracaine (25 μM) in SK‐N‐SH cells. Cellular invasion was evaluated by transwell‐Matrigel assay. (A) Representative images (10× objective) belonging to control (untreated) and tetracaine‐treated cells. (B) The percentage of invaded SK‐N‐SH cells was calculated by normalising to an untreated control group. Data bars in (B) denote means and SEM (*n* = 3).

On the other hand, SK‐N‐SH cells exhibited clear invasive behaviour and were tested in Matrigel‐based transwell invasion assays. Following 25 μM tetracaine treatment for 48 h, invasiveness was reduced significantly by ~20% (*p* < 0.01; Figure 3).

## 4 Discussion

The main findings of the study were as follows:

1. All 6 NB cell lines studied expressed VGSC mRNAs. Expression was heterogenous—*SCN8A* (Nav1.6) was expressed in all the cell lines, whereas *SCN5A* (Nav1.5) seemed expressed selectively in invasive cells.
2. At optimised concentrations, tetracaine had no effect on the viability and proliferative activities of the two contrasting model cells lines adopted: SH‐EP and SK‐N‐SH.
3. SH‐EP cells were motile but not invasive; tetracaine inhibited their lateral motility significantly by up to 35% over 48 h.
4. SK‐N‐SH cells were invasive and tetracaine inhibited their Matrigel invasiveness significantly by 20% over 48 h.

### 4.1 VGSC Expression in NB Cell Lines

According to the bioinformatics, the elevated expression of Nav1.5 in SK‐N‐SH cells (and other invasive cell lines) is noteworthy, as this channel has been linked to metastatic behaviour in breast and colorectal cancers [2, 10]. Its expression as a developmentally regulated neonatal splice variant was demonstrated earlier in NB‐1 cells [5]. Its selective enrichment in an aggressive NB cell line is consistent with a potential role in promoting tumour aggressiveness. Clinically, Nav1.5 may therefore represent both a biomarker of high‐risk NB and a candidate therapeutic target, where pharmacological inhibition of its activity could help to limit metastatic progression and improve patient outcome [3].

### 4.2 Effects of Tetracaine and Mechanistic Aspects

Tetracaine is a reversible blocker of VGSC activity, binding to the Na^+^ pore from the intracellular side of the channel protein [4]. It has been used extensively as a local anaesthetic clinically as well as to inhibit cellular VGSC activity experimentally [4, 11]. At the maximum concentration (100 μM) of tetracaine used here, it is likely that the VGSC currents would be blocked by some 50% [4]. In the two model NB cell lines adopted (SH‐EP and SK‐N‐SH), tetracaine had no effect on cell viability at concentrations up to 100 μM. This agrees with previous studies on NB, glioblastoma and oral squamous cell carcinoma cells showing cytotoxic effects of tetracaine only at concentrations greater than 100 μM [12]. We, therefore, adopted 50 and 25 μM of tetracaine, respectively, to study metastatic cell behaviours free from any possible effect on cell viability or proliferation over 48 h. Tetracaine inhibited the lateral motility SH‐EP cells significantly by 35%. Unlike SH‐EP, SK‐N‐SH cells were invasive and tetracaine inhibited the invasiveness by 20%. This was a true effect on invasion because, at the working concentration of tetracaine, neither proliferation nor cell viability were involved. These findings agree with previous work on multiple cancer types (especially breast, colon and prostate cancers) showing (i) that VGSC activity promotes metastatic cell behaviours and (ii) that local anaesthetics can block invasiveness [13, 14, 15]. On breast cancer MDA‐MB‐231 cells, for example, tetracaine had a significant suppressive effect on motility, invasion and adhesion [11]. A major mode of action of the cancer VGSC is in acidifying extracellular pH, leading to activation of proteolytic enzymes. Consistent with this, tetracaine was shown earlier to downregulate matrix metalloproteinases and upregulate tissue inhibitors of metalloproteinases [11]. VGSC activity can also affect the intracellular Ca^2+^ level via Na^+^/Ca^2+^ exchange (NCX) and together with the VGSC β‐subunits can lead to further remodelling of the cytoskeleton including the formation of actin‐rich invasive structures such as invadopodia [16]. Thus, VGSCs act as upstream regulators that integrate ionic signalling, adhesion dynamics, and gene expression to enhance migration and invasiveness of cancer cells [10].

### 4.3 Clinical Potential

In addition to tetracaine, several clinically approved VGSC modulators, originally developed for non‐oncological indications, have gained increasing attention for their potential anticancer properties. In particular, lidocaine, a widely used local anaesthetic, and ranolazine, an antianginal agent, have both demonstrated inhibitory effects on cancer cell proliferation, migration and invasion in preclinical including human studies [15, 17]. Furthermore, human trials suggested that perioperative administration of lidocaine can be associated with reduced cancer recurrence rates and longer patient survival [18] Another interesting candidate is ranolazine, which (i) binds to the same site intracellular site within the VGSC protein as local anaesthetics and (ii) blocks selectively the channel's persistent current leading to inhibition of metastasis with real‐world evidence suggesting a survival benefit [17]. Although such agents have been shown to produce anti‐cancer effects through VGSC inhibition dosages need to be carefully controlled since side effects are also possible (e.g., https://www.drugs.com/ranexa.html).

### 4.4 Limitations of the Study

Our study does suffer from two main limitations. First, we have not revealed the subtype(s) of the VGSCs present in the NB cells and if these are electrophysiological active. Second, we have not elucidated the possible signalling mechanisms associated with the anti‐invasive effect of tetracaine. Nevertheless, well‐established insights do exist for both limitations. Several NB cell lines have indeed been shown to express functional VGSCs, and their activity can induce changes in extracellular pH and intracellular Ca^2+^ with wide‐ranging downstream consequences, as noted in Section 4.2. We plan to probe deeper into these areas in future work.

## 5 Conclusion

Tetracaine has anti‐invasive effects on NB cells and, accordingly, may ultimately be repurposed as a safe clinical agent against NB especially its aggressive forms.

## Funding

This study was supported by Pro Cancer Research Fund (PCRF).

## Conflicts of Interest

M.B.A.D. holds shares in Celex Oncology Innovations Ltd., which aims to develop ion channel modulators as cancer drugs. The other authors declare no conflicts of interest.

## Data Availability

The data that support the findings of this study are available from the corresponding author upon reasonable request.
